# Effects of Exercise on Cognitive Function in People with Intellectual Disabilities: A Systematic Review and Meta-Analysis of Randomized Controlled Trials

**DOI:** 10.3390/brainsci15111203

**Published:** 2025-11-07

**Authors:** Yifan Zhang, Shiyan Zhang, Gen Li, Yuanyuan Lv, Lingxiao He, Laikang Yu

**Affiliations:** 1 Beijing Key Laboratory of Sports Performance and Skill Assessment, Beijing Sport University, Beijing 100084, China; 18738314013@163.com (Y.Z.); sunflowerlyy@bsu.edu.cn (Y.L.); 2 Department of Strength and Conditioning Assessment and Monitoring, Beijing Sport University, Beijing 100084, China; 3 School of Sport Sciences, Beijing Sport University, Beijing 100084, China; 17634977464@163.com; 4 School of Physical Education & Sports Science, South China Normal University, Guangzhou 510631, China; li287270242@163.com; 5 China Institute of Sport and Health Science, Beijing Sport University, Beijing 100084, China; 6 School of Public Health, Xiamen University, Xiamen 361005, China

**Keywords:** exercise, cognitive function, executive function, intellectual disabilities, systematic review, meta-analysis

## Abstract

**Objectives**: This study aimed to evaluate the effects of exercise on cognitive function in individuals with intellectual disabilities (ID) and to identify the optimal exercise prescription. **Methods**: A comprehensive search of PubMed, Web of Science, EBSCO, Cochrane, and Scopus was conducted through 13 May 2025. The Cochrane risk assessment tool was used to evaluate the methodological quality of the included studies. **Results**: Twenty studies fulfilled the inclusion criteria, of which six were rated as high quality and eleven as moderate quality. A meta-analytic synthesis of 14 eligible studies demonstrated that exercise elicited a significant improvement of cognitive function in ID patients (Hedges’ *g* = 0.85, *p* < 0.001), with the greatest effect observed for cognitive speed (Hedges’ *g* = 0.93, *p* < 0.01). Subgroup analyses indicated that interventions lasting ≥12 weeks (Hedges’ *g* = 0.92, *p* < 0.001), performed <3 times per week (Hedges’ *g* = 1.22, *p* < 0.01), with sessions ≥60 min (Hedges’ *g* = 1.91, *p* < 0.01), and >180 min per week in total (Hedges’ *g* = 3.10, *p <* 0.01) yielded the most pronounced benefits. Adolescents with ID exhibited greater cognitive gains (Hedges’ *g* = 1.01, *p* < 0.001). **Conclusions**: Exercise significantly improved cognitive function in ID patients. Our findings suggested that ID patients may benefit from exercise sessions lasting at least 60 min, performed fewer than three times per week, and sustained for at least 12 weeks. Achieving an exercise target of more than 180 min per week may further enhance cognitive function. Moreover, younger ID patients may experience greater improvements in cognitive function. Future studies should focus on standardizing exercise protocols and cognitive assessment tools to ensure consistency and comparability of findings in this population.

## 1. Introduction

Intellectual Disability (ID) is a neurodevelopmental disorder characterized by substantial limitations in intellectual functioning and adaptive behavior, typically manifesting during the developmental period [1]. The etiology of ID is multifactorial, involving genetic disorders, maternal illnesses, poverty, maternal malnutrition, exposure to hazardous activities, maternal substance abuse, and advanced parental age [2]. Globally, ID affects approximately 1% of the population, with around 85% of cases classified as mild [3]. Prevalence is notably higher in low- and middle-income countries compared to high-income settings, with the highest incidence observed among children and adolescents [4]. Cognitive impairment is a defining feature of ID, resulting in difficulties in learning, reasoning, problem-solving, and everyday activities, including communication, social interaction, and self-care skills [5]. These deficits substantially affect quality of life, educational attainment, and employment prospects. Furthermore, the rate of cognitive development is generally slower in individuals with ID than in the general population [6]. Consequently, early educational and targeted interventions, such as speech therapy and occupational therapy, have been recommended to mitigate developmental delays [7,8]. Traditional rehabilitation strategies for ID encompass medical care, cognitive training, educational interventions, early interventions programs, behavioral therapies, and supportive measures [9].

In recent years, exercise has gained recognition as a promising non-pharmacological approach for enhancing cognitive function in the general population. Increasing attention has been directed toward its potential to improve cognitive functions in individuals with ID. Exercise has been shown to have a positive impact on brain health, possibly by stimulating neuroplasticity, enhancing blood flow to the brain, and modulating neuro-chemical processes such as the release of brain-derived neurotrophic factor (BDNF) [10,11]. Furthermore, exercise can reduce stress and anxiety, which may improve cognitive performance indirectly by enhancing overall mental well-being [12]. However, given the distinctive nature of functional impairments in this population, the cognitive benefits of exercise may not mirror those observed in typically developing individuals. Previous studies examining exercise interventions in ID have reported mixed findings. For instance, Sriharisukesh et al. [13] observed significant improvements in executive functions following a 5-week yoga training program, while Wang et al. [14] documented enhancements in both executive and memory functions after a 12-week badminton intervention. These results suggest that exercise may represent a viable strategy for improving cognitive abilities in ID. In contrast, Jacinto et al. [15] found that a 24-week program combining indoor and outdoor activities increased physical activity levels but did not yield significant cognitive gains. Such inconsistencies may stem from differences in exercise type, intensity, duration, and the cognitive domains assessed.

A previous meta-analysis investigated the effects of exercise on cognitive functions in children and adolescents with ID and concluded that physical activity exerted a positive influence on overall cognitive outcomes [16]. However, this analysis had several important limitations. It included participants with various developmental disorders, not solely individuals with ID, and did not restrict its inclusion criteria to randomized controlled trials (RCTs). As a result, the findings lacked specificity for the ID population and were affected by methodological inconsistencies. Additionally, the absence of RCTs in the included studies reduces the methodological rigor and limits the ability to draw definitive causal conclusions. In contrast, the present study focuses exclusively on individuals with ID and only includes RCTs, ensuring a more robust, targeted, and methodologically rigorous evaluation of exercise-induced cognitive benefits. Furthermore, previous research has been constrained by significant methodological heterogeneity, including differences in cognitive assessment tools and exercise protocols, which complicates cross-study comparisons and synthesis of results. By addressing these limitations, our study provides a clearer, more precise understanding of the cognitive benefits of exercise in the ID population.

To address these limitations, the present systematic review and meta-analysis focuses exclusively on RCTs evaluating the effects of exercise on cognitive functions in ID patients. The primary aim is to determine whether robust evidence supports the use of exercise as an effective intervention to enhance cognitive function in this population and to identify the characteristics of exercise programs most conducive to achieving such benefits. It was hypothesized that exercise would produce significant enhancements in executive and memory functions in individuals with ID, with more pronounced effects observed in adolescents.

## 2. Materials and Methods

This systematic review and meta-analysis was conducted in accordance with the Preferred Reporting Items for Systematic Reviews and Meta-Analyses (PRISMA) guidelines [17]. The study protocol was registered in PROSPERO (CRD420251060322).

### 2.1. Search Strategy

A comprehensive search was performed across PubMed, Web of Science, EBSCO, Cochrane, and Scopus to identify all relevant studies up to 13 May 2025. Search terms included combinations of keywords and Medical Subject Headings (MeSH) related to intellectual disability, exercise, and cognitive function (Table S1). In addition, the reference lists of relevant reviews or meta-analyses were manually screened to identify further eligible studies. Two reviewers (Y.Z. and S.Z.) independently conducted the search and selection process following a standardized protocol. Disagreements were resolved through discussion with a third reviewer (L.Y.) until consensus was reached.

### 2.2. Eligibility Criteria

Studies were included if they met the following criteria: (1) RCT design; (2) inclusion of both intervention and control groups; (3) participants diagnosed with ID; and (4) cognitive function assessed as an outcome measure.

Studies were excluded if they: (1) were published in languages other than English; (2) used animal models; (3) were review articles or conference abstracts; or (4) lacked full-text availability.

### 2.3. Data Extraction

Two reviewers (Y.Z. and S.Z.) independently extracted data, including: (1) study characteristics (first author, publication year, sample size); (2) intervention details (duration, frequency, session length); (3) participant characteristics [age, intelligence quotient (IQ), body mass index (BMI)]; and (4) treatment effects [mean and standard deviation (SD) for changes in cognitive function from baseline to post-intervention].

### 2.4. Methodological Quality Assessment

The methodological quality of included studies was evaluated using the Cochrane Risk of Bias (RoB 1) tool, which evaluates seven items: random sequence generation (selection bias), allocation concealment (selection bias), blinding of participants and personnel (performance bias), blinding of outcome assessment (detection bias), incomplete outcome data (attrition bias), selective reporting (reporting bias) and other biases. Each domain was rated as “low risk”, “unclear risk”, or “high risk” of bias [18]. Two reviewers (Y.Z. and S.Z.) independently conducted the assessment, resolving any discrepancies through discussion with a third reviewer (L.Y.).

### 2.5. Statistical Analysis

As multiple cognitive outcomes were often reported within studies, it was inappropriate to assume that the results of each study were independent or to estimate them as equivalent. Consequently, a three-level restricted maximum likelihood random effects model was applied using the metafor package in R [19], following the computational framework described by Assink et al. [20]. This model accounts for the dependency of effect sizes by estimating within-study (level 2) and between-study (level 3) variance.

The primary effect size was expressed as mean ± SD, standardized using Hedges’ *g* with correlation r = 0.5 to compare pre-to-post changes in cognitive function between exercise and control groups. Positive values indicate superior improvement in the exercise group. Effect size magnitude was interpreted as small (*g* = 0.2–0.5), medium (*g* = 0.5–0.8), or large (*g* ≥ 0.8) [21,22]. Heterogeneity was quantified using the I^2^ statistic, with thresholds of <25% (no significant heterogeneity), 25–50% (low), 50–75% (moderate), and >75% (high) [23,24].

A key limitation in synthesizing the results of these studies is the methodological heterogeneity across studies, particularly in the cognitive assessment tools used. Cognitive function is often measured using a variety of tools, including tests of executive function (EF), memory, and cognitive speed. This variation in tools may limit the comparability of results across studies and introduce residual heterogeneity in the effect sizes. Differences in the sensitivity and specificity of these tools, as well as their respective domains of assessment, may contribute to inconsistent findings and affect the generalizability of the results. To account for this, subgroup analyses examined the effects of exercise by cognitive domain (EF, memory, cognitive speed) [25,26], session duration (<60 min, ≥60 min), frequency (<3 times per week, ≥3 times per week), intervention length (<12 weeks, ≥12 weeks), weekly time (≤180 min, >180 min), and participant age (adolescents, adults).

Forest and funnel plots were generated in R version 4.2.2 (R Foundation for Statistical Computing, Vienna, Austria). Egger’s test, sensitivity analyses, and meta-regression were conducted in Stata 18. A two-sided *p* < 0.05 was considered statistically significant.

## 3. Results

### 3.1. Study Selection

**Figure 1:**
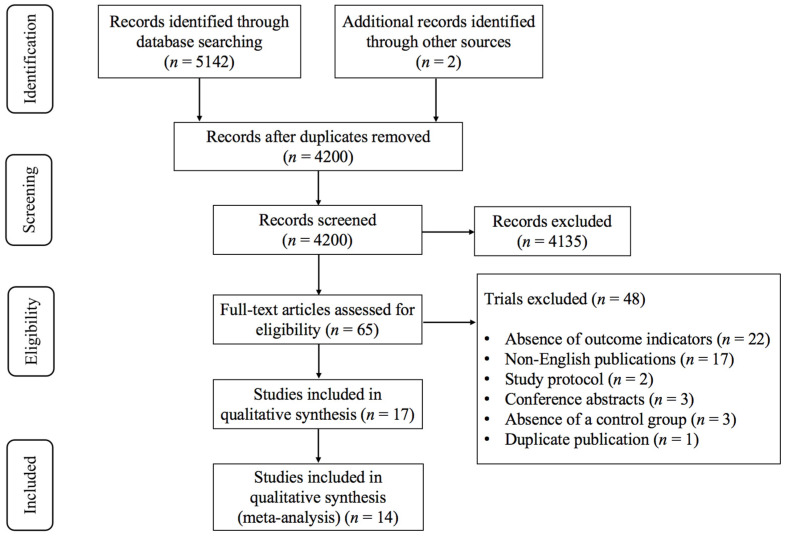
PRISMA flowchart of study selection.

As shown in Figure 1, a total of 5142 records were retrieved from databases, with 2 additional records identified from other sources. After removing duplicates, 4200 unique records remained. Screening of titles and abstracts excluded 4135 records that did not meet the inclusion criteria. Full-text review of 65 studies led to the exclusion of 45 studies for the following reasons (Table S2): absence of outcome indicators (*n* = 22), non-English publications (*n* = 17), study protocol (*n* = 2), conference abstracts (*n* = 3), absence of a control group (*n* = 3), and duplicate publication (*n* = 1). Ultimately, 17 studies [13,14,15,27,28,29,30,31,32,33,34,35,36,37,38,39,40] met the eligibility criteria and 14 studies [13,14,15,27,28,29,30,31,32,33,34,35,36,37] were selected for further analysis.

### 3.2. Characteristics of the Included Studies

Participant and intervention details are presented in Table S3. A total of 674 participants were included across 24 exercise groups, and 395 participants in 17 control groups. Among these 17 studies, 4 were three-arm trials [27,28,31,33], and 1 was a multi-arm trial [39]. Ten studies [27,28,29,30,31,32,33,37,39,40] focused on adolescents with ID, whereas the remaining seven investigated adults with ID [13,14,15,34,35,36,38]. Ten studies [13,14,15,29,30,32,33,36,37,38] reported gender information, while seven [27,28,31,34,35,39,40] did not provide detailed gender data. Eight studies [14,27,29,30,33,34,35,40] reported participants’ IQ levels, and nine [13,15,28,31,32,36,37,38,39] did not report this key information. Fourteen studies [13,14,15,27,28,30,31,32,34,35,36,37,38,40] examined processing speed, 14 [13,14,15,27,28,29,30,31,32,33,35,36,38,40] investigated executive function, and 12 [14,15,27,29,30,31,33,35,36,38,39,40] focused on memory. All studies employed diversified training interventions, which included balance training, trampoline exercises, running, coordination training, aquatic training, badminton, yoga, swimming, basketball, and other intervention forms. The total duration of interventions ranged from 5 to 128 weeks, with session durations varying from 30 min to 180 min. The frequency of intervention per week ranged from 2 to 7 times. Due to the variability in the frequency and duration of interventions across studies, we calculated the weekly minutes of intervention based on the duration per session and frequency in the included studies. Weekly intervention minutes ranged from 60 to 900 min.

### 3.3. Meta-Analysis Results

**Figure 2:**
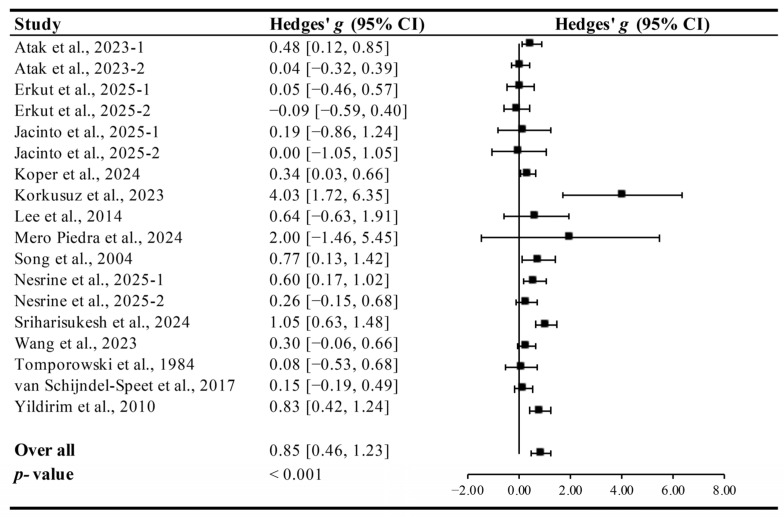
Meta-analysis results on the effects of exercise on cognitive function in ID patients [13,14,15,27,28,29,30,31,32,33,34,35,36,37].

Pooled analysis indicated that exercise significantly improved cognitive function in ID patients, with a large effect size (Hedges’ *g* = 0.85, 95% CI 0.46 to 1.23, *p* < 0.001, Figure 2) and moderate heterogeneity (I^2^ = 46.85%).

### 3.4. Meta-Regression Analysis

As shown in Table S4, the meta-regression analysis indicates that cognitive function is significantly correlated with weekly time (*p* < 0.0001) and session duration (*p* = 0.025). However, there were no significant correlations between cognitive function and cognitive domain (*p* = 0.453), intervention duration (*p* = 0.589), frequency (*p* = 0.261), or participant age (*p* = 0.483).

### 3.5. Subgroup Analysis

**Figure 3:**
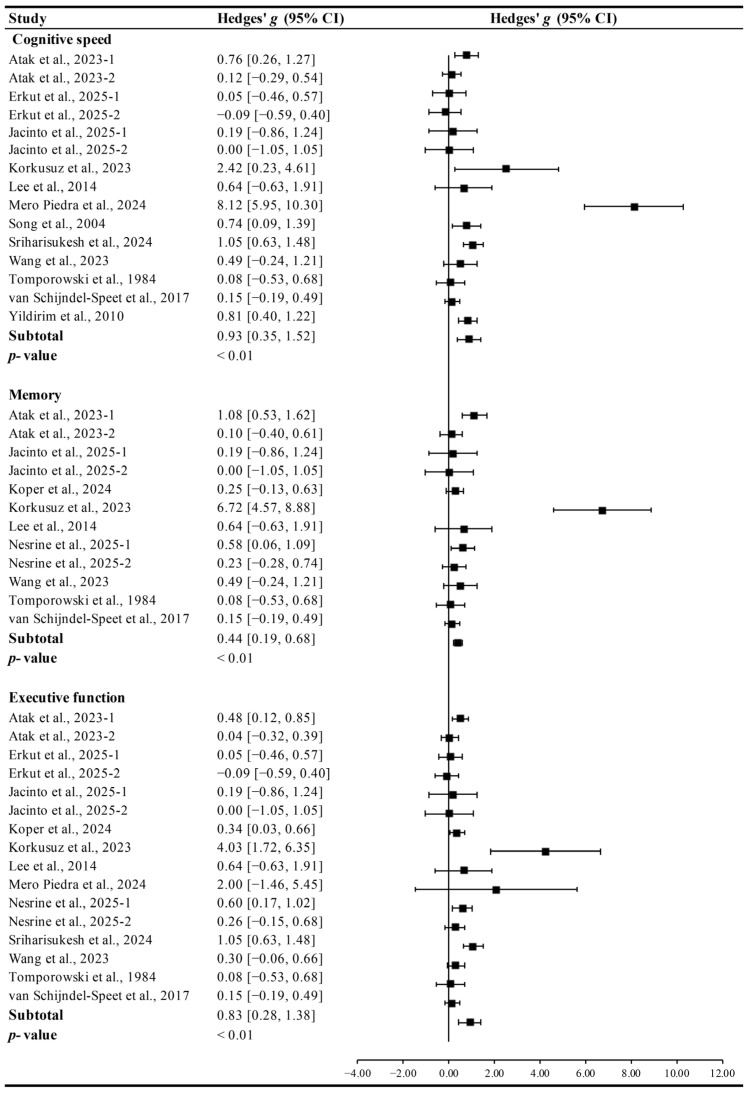
Meta-analysis results on the effects of exercise on various cognitive domains in ID patients [13,14,15,27,28,29,30,31,32,33,34,35,36,37].

Exercise produced significant improvements in EF (Hedges’ *g* = 0.83, 95% CI 0.28 to 1.38, *p* < 0.01, I^2^ = 47.55%), cognitive speed (Hedges’ *g* = 0.93, 95% CI 0.35 to 1.52, *p* < 0.01, I^2^ = 46.38%), and memory in ID patients (Hedges’ *g* = 0.44, 95% CI 0.19 to 0.68, *p* < 0.01, I^2^ = 24.19%, Figure 3), with the greatest effects observed for cognitive speed.

**Figure 4:**
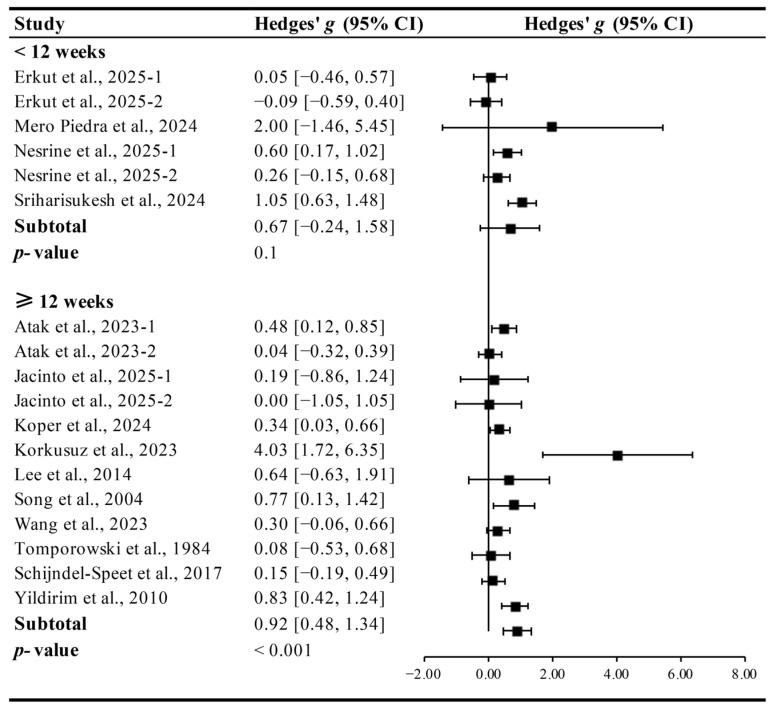
Meta-analysis results on the effects of intervention duration on cognitive function in ID patients [13,14,15,27,28,29,30,31,32,33,34,35,37].

Interventions conducted for ≥12 weeks (Hedges’ *g* = 0.91, 95% CI 0.48 to 1.34, *p* < 0.001, I^2^ = 46.60%) had a significant effect on improving cognitive function in ID patients, whereas those <12 weeks were not significant (Hedges’ *g* = 0.67, 95% CI −0.24 to 1.58, *p* = 0.10, I^2^ = 47.55%, Figure 4).

**Figure 5:**
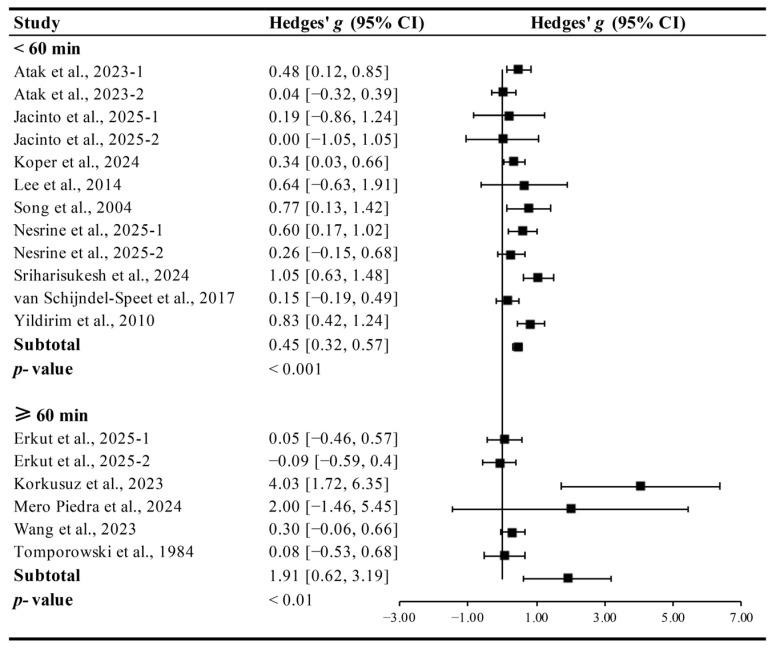
Meta-analysis results on the effects of session duration on cognitive function in ID patients [13,14,15,27,28,29,30,31,32,33,34,35,36,37].

Interventions conducted for <60 min (Hedges’ *g* = 0.45, 95% CI 0.32 to 0.57, *p* < 0.001, I^2^ = 14.75%) and ≥60 min per session (Hedges’ *g* = 1.91, 95% CI 0.62 to 3.19, *p* < 0.01, I^2^ = 49.12%, Figure 5) significantly improved cognitive function in ID patients, but longer sessions yielded larger effects.

**Figure 6:**
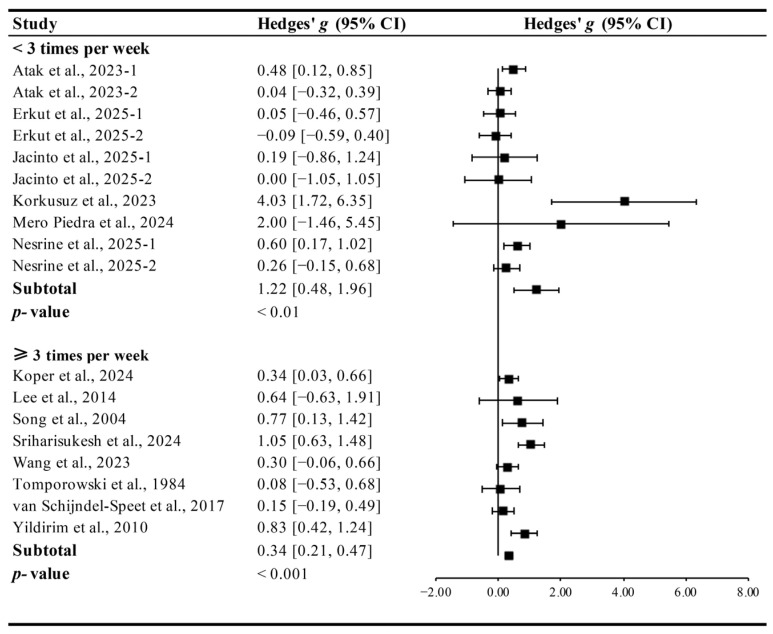
Meta-analysis results on the effects of intervention frequency on cognitive function in ID patients [13,14,15,27,28,29,30,31,32,33,34,35,36,37].

Interventions conducted for <3 times per week (Hedges’ *g* = 1.22, 95% CI 0.48 to 1.96, *p* < 0.01, I^2^ = 48.51%) and ≥3 times per week (Hedges’ *g* = 0.49, 95% CI 0.33 to 0.65, *p* < 0.001, I^2^ = 14.63%, Figure 6) significantly improved cognitive function in ID patients, with lower-frequency programs yielded greater effects.

**Figure 7:**
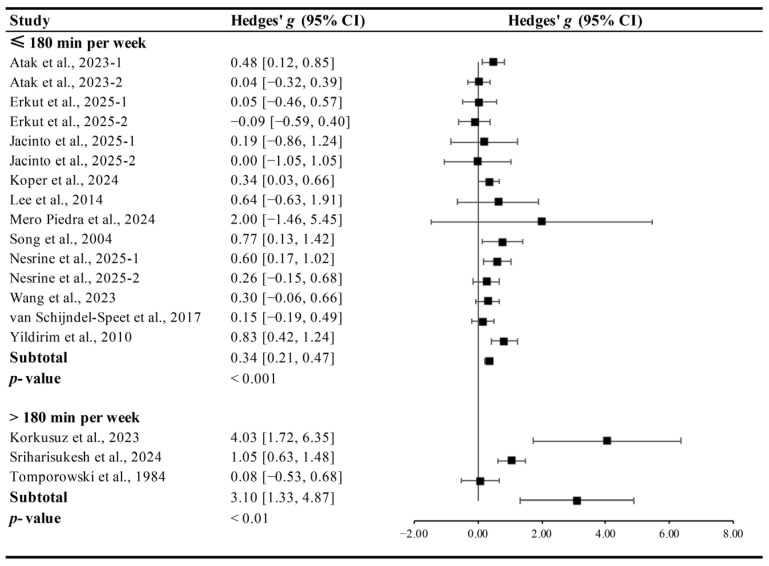
Meta-analysis results on the effects of weekly time on cognitive function in ID patients [13,14,15,27,28,29,30,31,32,33,34,35,36,37].

Interventions conducted for ≤180 min per week (Hedges’ *g* = 0.34, 95% CI 0.21 to 0.47, *p* < 0.001, I^2^ = 21.38%) and >180 min per week (Hedges’ *g* = 3.10, 95% CI 1.33 to 4.87, *p* < 0.01, I^2^ = 49.04%, Figure 7) significantly improved cognitive function in ID patients, though higher volumes produced larger effects.

**Figure 8:**
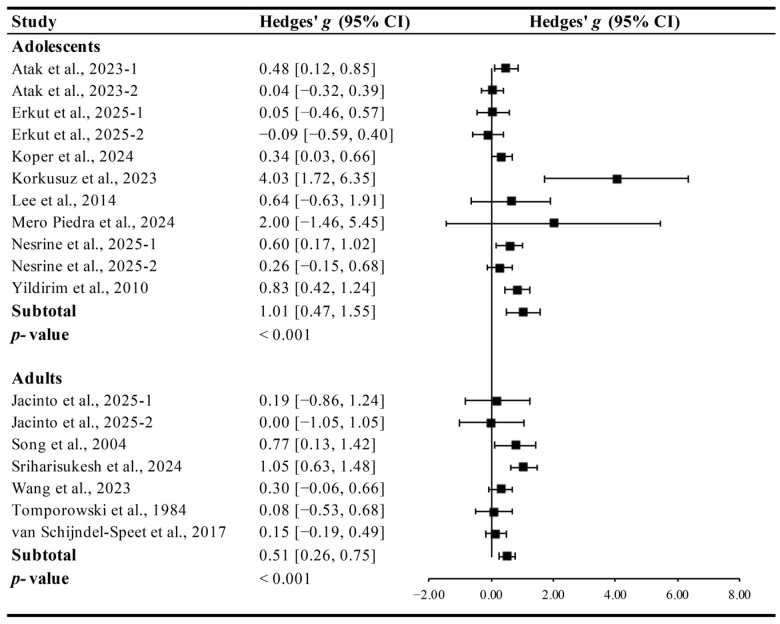
Meta-analysis results on the effects of exercise on cognitive function in adolescents and adults with ID [13,14,15,27,28,29,30,31,32,33,34,35,36,37].

Exercise significantly improved cognitive function in both adolescents (≤18 years, Hedges’ *g* = 1.01, 95% CI 0.47 to 1.55, *p* < 0.001, I^2^ = 47.90%) and adults (>18 years, Hedges’ *g* = 0.51, 95% CI 0.26 to 0.75, *p* < 0.001, I^2^ = 20.85%, Figure 8), with greater effects observed in adolescents.

### 3.6. Risk of Bias

The quality of the included studies was assessed using the RoB 1 tool across six domains: selection bias, performance bias, detection bias, attrition bias, reporting bias, and other biases. Trials with high risk for randomization or allocation concealment were classified as low quality; those with low risk in these domains and no high risk elsewhere were considered; all others were rated moderate [41]. Of the 17 studies, six [13,14,27,35,36,37] were rated high quality and 11 [15,28,29,30,31,32,33,34,38,39,40] moderate quality (Figure S1). Furthermore, according to the GRADE system, all outcomes were rated as having high certainty.

### 3.7. Publication Bias

Visual inspection of the funnel plot (Figure S2) and Egger’s test (k = 63 effects from 14 studies, t = −3.92, *p* = 0.0001, Table S6) indicated potential publication bias. Trim-and-fill analysis yielded an adjusted effect size identical to the unadjusted estimate (Hedges’ g = 0.618, 95% CI, 0.416 to 0.820, *p* < 0.0001), with no imputed studies.

### 3.8. Sensitivity Analysis

Sensitivity analysis confirmed the robustness of findings; exclusion of any single study did not materially alter the direction and magnitude of the pooled effect (Figure S3).

## 4. Discussion

### 4.1. Main Findings

This study evaluated the effects of exercise on cognitive function in ID patients by synthesizing data from 17 studies involving a total of 1069 participants. The results demonstrated that exercise significantly improved cognitive function, particularly in cognitive speed and EF. Subgroup analyses revealed that interventions of at least 12 weeks, conducted less than three times per week, with each session lasting a minimum of 60 min and a total weekly duration exceeding 180 min, yielded greater cognitive gains. Notably, these benefits were more pronounced in participants aged 18 years or younger.

### 4.2. Effect of Exercise on Cognitive Function in ID Patients

Extensive evidence supports the beneficial effects of exercise on both physical health and cognitive function in individuals with ID [42]. However, findings remain somewhat inconsistent, likely due to inter-individual variability and differences in exercise protocols. Exercise may enhance cognitive function through multiple mechanisms, with outcomes influenced by participant characteristics.

From the perspective of motor reserve, exercise can enhance neural adaptability, enabling the brain to better cope with neurological damage or degeneration through reorganization of neural networks and functional compensation. Motor reserve is defined as the capacity to recruit intact brain regions to compensate for impaired areas, thereby preserving or restoring cognitive function [43]. From a neuroplasticity standpoint, exercise elevates levels of BDNF, a key protein involved in neuronal growth and synaptic plasticity. This upregulation promotes synaptogenesis, ultimately enhancing memory and learning abilities [31,44,45,46].

Direct neurobiological evidence within the RCTs included in this meta-analysis was limited. In one trial involving adults with intellectual disability, aquatic exercise led to increases in serum BDNF and vascular endothelial growth factor (VEGF), and the change in BDNF correlated with cognitive gains, providing in-sample support that exercise-induced upregulation of neurotrophic and angiogenic pathways accompanies cognitive improvement in ID [31]. By contrast, all other included RCTs assessed cognition but did not collect mechanistic biomarkers or imaging; therefore, discussion of putative pathways (e.g., synaptic plasticity, hippocampal/anterior cingulate modulation, and white-matter integrity) remains inferential and should be interpreted as contextual rather than trial-level evidence.

Previous research demonstrates that exercise enhances cognitive performance by improving white matter integrity and activating key brain regions such as the hippocampus and prefrontal cortex [47,48,49]. Additional mechanisms include the promotion of neurogenesis, cell survival, synaptogenesis, and synaptic plasticity [50,51]. The cognitive benefits may vary depending on exercise modality [52,53]. Coordination training and sensory integration activities provide multimodal stimulation to the nervous and sensory systems, effectively activating neural networks associated with attention control and executive functions [54]. Endurance training may contribute indirectly by improving brain metabolism and vascular health [55]. In this meta-analysis, it remains unclear whether the included interventions targeted specific cognitive domains or were adapted to participants’ functional abilities.

Social and emotional factors also contribute to cognitive enhancement. Exercise can improve emotional well-being, alleviate symptoms of anxiety and depression, and foster social support through interpersonal engagement [56]. These psychosocial benefits may indirectly promote cognitive gains. However, cognitive outcomes are moderated by factors such as age, sex, baseline cognitive status, health conditions, and genetic background, emphasizing the importance of personalized intervention strategies [57].

### 4.3. Effects of Different Exercise Modalities on Cognitive Function in ID Patients

To investigate the specific cognitive abilities influenced by exercise, cognitive function was classified into three domains: memory, cognitive speed, and EF. Twelve studies assessed memory using instruments such as the Wechsler Intelligence Scale for Children-Revised, Mini-Mental State Examination, Fifth Edition of Stanford–Binet Intelligence Subtests, Benton Visual Retention Test, Korean Western Aphasia Battery, Digit Span Task, Corsi Block-Tapping Test, 1-Back Test, Stanford–Binet Intelligence Scales, Stanford-Binet (L-M), Leiter International Performance Scale, DMR Cognitive Subscale, and the Adaptive Behaviour Scale. Fourteen studies evaluated cognitive speed using assessments including the MOXO Attention Test, Wechsler Intelligence Scale for Children-Revised, Frostig Visual Perception Test, Mini-Mental State Examination, d2 Test of Attention, Korean Western Aphasia Battery, Vigilance Tasks, Premotor RT, Motor RT, Wall Toss Test, Finger Tapping Test, Six-Letter Cancellation Task, Task Switching Test, Stanford–Binet Intelligence Scales, Leiter International Performance Scale, DMR Cognitive Subscale, Bruininks–Oseretsky Test of Motor Proficiency, and the Stanford Binet Test. Fourteen studies assessed EF using tests such as the MOXO Attention Test, Wechsler Intelligence Scale for Children-Revised, Frankfurter Attention Test, Frostig Visual Perception Test, Mini-Mental State Examination, the Fifth Edition of Stanford–Binet Intelligence Subtests, d2 Test of Attention, Korean Western Aphasia Battery, Vigilance Tasks, Distractor Interference Tasks, Response Inhibition Tasks, the Zazzo Two-Sign Barrage Test, Wall Toss Test, Finger Tapping Test, Six-Letter Cancellation Task, Stroop Test, Task Switching Test, Stanford-Binet (L-M), Leiter International Performance Scale, DMR Cognitive Subscale.

Subgroup analyses confirmed that exercise significantly improved cognitive speed and EF, with findings consistent with a prior meta-analysis, showing that in adolescents with ID, improvements were particularly evident in working memory updating, sustained attention, and attentional allocation [16]. Experimental evidence suggests that even a single 30 min bout of running can alter frontal cortex activity [58], and aerobic exercise can increase volumes of brain regions associated with executive processing [59,60]. Since cognitive speed, EF, and memory are interrelated [61], gains in one domain may facilitate improvements in others. Exercise appears particularly effective for enhancing memory in neurodegenerative populations [25,26,62], although methodological heterogeneity may underestimate these effects in ID.

Intervention characteristics, including duration, frequency, session length, and weekly time, moderate cognitive benefits [63]. In this study, all interventions employed multicomponent training, integrating diverse exercise types to target multiple physical capabilities, which may better meet the needs of ID patients, given the wide range of individual differences in ID patients [64]. Enjoyable intervention elements may increase adherence [65]. While direct comparisons with single-modality programs remain limited, multicomponent training may achieve higher compliance.

Intervention duration exerted a clear dose–response relationship: interventions lasting ≥12 weeks significantly enhanced cognitive function, while shorter interventions did not [66]. This temporal threshold aligns with neuroplastic evidence showing that at least 12 weeks of moderate-intensity exercise are required to elicit detectable hippocampal volume expansion (~2%), while shorter durations do not reliably induce comparable structural changes in striatal or posterior cingulate regions [67].

Subgroup analysis revealed that both low-frequency (<3 times per week) and higher-frequency (≥3 times per week) interventions produced significant cognitive gains, while the former elicited larger effect sizes. This pattern concurs with the U.S. Department of Health and Human Services guidelines for ID patients, which prescribe at least two weekly sessions of moderate-intensity, multi-muscle-group exercise for adults and three combined aerobic, muscle- and bone-strengthening sessions for children and adolescents, adjusted to individual capacity [68]. ID patients generally exhibit lower cardiorespiratory fitness and muscular endurance, predisposing them to earlier neuromuscular fatigue [68]; marked heterogeneity in exercise tolerance further arises from variable baseline fitness and prevalent comorbidities. Consequently, individualized, periodized training is required to balance stimulus sufficiency with recovery demands. Although the cognitive benefits of exercise in this population are well established, high-frequency regimens risk central and peripheral fatigue that may attenuate neuroplastic adaptations [69]; deliberate fatigue-monitoring is therefore essential.

While there is strong evidence supporting exercise-induced cognitive benefits, further research is needed to determine the optimal session duration for individuals with ID. Moderate-intensity activity increases serum BDNF levels in healthy adults, and longer high-intensity sessions, such as 40 min, have been shown to elicit greater BDNF release than shorter sessions [70,71]. In our study, exercise sessions lasting ≥60 min were associated with improved cognitive outcomes, likely due to the greater sensorimotor and cognitive challenges posed by longer sessions. Prolonged sessions provide more comprehensive stimulation, which may be especially beneficial for children, a group with heightened neuroplasticity [72]. However, care should be taken with excessively long sessions, as they could lead to fatigue that may temporarily diminish cognitive benefits [69]. Given the individual variability among participants, further research is needed to better understand how session duration can be optimized for individuals with ID. Intervention strategies should be tailored to meet the specific needs of each individual.

According to the 2020 World Health Organization (WHO) Guidelines on Physical Activity and Sedentary Behavior, adults should engage in 150–300 min of moderate-intensity or 75–150 min of vigorous-intensity aerobic activity per week, or an equivalent combination of both [73]. Our findings suggest that exercise duration exceeding 180 min per week produces the largest effect sizes, reinforcing the importance of session duration and supporting the recommendation that each exercise session should last at least 60 min. Based on the frequency analysis, we recommend no more than 3 sessions per week, with each session lasting at least 60 min, to maintain a weekly exercise duration of over 180 min, while mitigating fatigue-related decrements in performance.

Notably, in the >180 min per week subgroup the pooled effect was Hedges’ *g* = 3.10, an unusually large estimate for cognitive outcomes. Influence analysis (leave-one-out) identified Korkusuz et al. [30] as highly influential; removing this study reduced the pooled effect to g = 0.82, preserving the direction and statistical significance. This suggests that extreme values from individual trials can inflate subgroup estimates. Accordingly, we interpreted the >180 min per week finding as suggestive rather than definitive, given heterogeneity in study designs and outcome instruments and the potential for measurement bias. Even so, the overall pattern indicates that weekly exercise volumes exceeding 180 min are associated with greater cognitive gains, warranting confirmation in trials with standardized cognitive measures and detailed dosing reports.

Given the pronounced inter-individual variability observed in individuals with ID, intervention designs must account for individual differences and employ personalized approaches. Previous research has indicated that excessively long single exercise sessions may induce fatigue, which could potentially reduce the efficacy of the intervention [74]. Therefore, compared to higher-frequency interventions, strategies that appropriately extend exercise duration while managing fatigue may be more suitable and effective for this population [75].

### 4.4. Effects of Exercise on Cognitive Function in Adolescents and Adults with ID

Age-stratified analyses underscore the significance of developmental timing, particularly in adolescents with ID, where exercise interventions show marked effects on cognitive function. Adolescence, a critical period for rapid physical and neurological maturation, is characterized by heightened neuroplasticity, making the nervous system more responsive to exercise [76]. Exercise increases BDNF levels, promotes neuronal growth, and enhances synaptic plasticity, thereby improving cognition [77]. This period, often referred to as the “neuroplasticity window”, enables the brain to adapt more easily to external stimuli, which may explain why adolescents with ID exhibit a stronger response to exercise [76].

The concept of cognitive reserve further clarifies why younger individuals with ID may benefit more from exercise. Due to their higher neuroplastic potential, adolescents are better able to compensate for cognitive impairments through exercise-induced neural adaptations. Developmental research suggests that younger individuals are more adept at forming new neural connections and reorganizing existing ones to meet cognitive and physical challenges [78]. Early intervention during childhood and adolescence can take advantage of these developmental windows, offering long-term cognitive benefits. Implementing structured exercise at this stage helps establish a stronger cognitive foundation, optimize brain function, and potentially delay cognitive decline in adulthood, highlighting the importance of early, targeted interventions for adolescents with ID.

### 4.5. Limitations

This study has several limitations worth considering. First, implementing blinding procedures in RCTs involving exercise interventions is inherently challenging, and subjective factors may introduce bias in quality assessments. Second, the relatively small sample sizes of most included trials may reduce statistical power and limit the generalizability of the findings. Third, the use of different cognitive scales across studies may lead to outcome variability; future research could benefit from using standardized cognitive assessment tools. Fourth, most of the included studies did not provide detailed information on exercise intensity or adherence, and few incorporated objective measures to monitor training load, thereby limiting our ability to clarify the dose–response relationship between exercise intensity and cognitive function. Fifth, the majority of trials lacked long-term follow-up assessments, preventing evaluation of the sustainability of cognitive benefits. Additionally, multi-component training was the primary intervention in all studies, making it difficult to isolate the specific effects of individual exercise modalities on cognitive outcomes. Furthermore, many studies did not report participant details regarding the severity of ID, which restricts our ability to explore how exercise affects individuals with varying levels of impairment. Finally, insufficient reporting on comorbidities limits our ability to investigate potential associations between comorbid conditions and cognitive function.

It is also worth noting that several outcomes in this meta-analysis yielded very large effect sizes. Such values, while statistically valid, may indicate potential overestimation or publication bias due to small sample sizes, heterogeneous methodologies, or selective reporting. Therefore, these results should be interpreted with caution, and future large-scale, preregistered trials are needed to confirm the robustness and reproducibility of these findings. Future research should therefore adopt larger, well-controlled RCT designs with standardized exercise protocols, objective intensity monitoring, extended follow-up periods, and comprehensive participant characterization to strengthen the evidence base.

## 5. Conclusions

Exercise was found to significantly improve cognitive function in ID patients. The findings of this study suggested that ID patients may benefit from engaging in exercise sessions less than three times per week, with each session lasting at least 60 min, over a minimum duration of 12 weeks. Achieving a weekly exercise target of at least 180 min may further enhance cognitive outcomes. While both exercise duration and frequency demonstrated effectiveness, the impact on cognitive function was particularly pronounced in younger individuals, indicating that earlier intervention may yield greater cognitive benefits.

These findings underscored the importance of considering individual patient characteristics, such as age, when designing exercise interventions to optimize cognitive improvements in ID patients. Further research with larger sample sizes and more targeted subgroup analyses is necessary to confirm the specific benefits of exercise across different age groups and varying levels of ID.

## Data Availability

All data generated or analyzed during this study are included in the article/Supplementary Material.

## Supplementary Materials

The following supporting information can be downloaded at: https://www.mdpi.com/article/10.3390/brainsci15111203/s1, Figure S1: Results of Cochrane risk of bias tool; Figure S2: Funnel plot; Figure S3: Sensitivity analysis results; Table S1: Search strategy; Table S2: Excluded studies list; Table S3: Characteristics of studies included in this meta-analysis; Table S4: Results of meta-regression analysis; Table S5: GRADE summary of evidence; Table S6: Results of Egger’s test.

## Abbreviations

The following abbreviations are used in this manuscript:

ID	Intellectual disabilities
RCTs	Randomized controlled trials
IQ	Intelligence quotient
BMI	Body mass index
SD	Standard deviation
EF	Executive function
CI	Confidence intervals
BDNF	Brain-derived neurotrophic factor
WHO	The World Health Organization

## References

[1] Schalock R.L., Luckasson R., Tassé M.J. (2021). An overview of intellectual disability: Definition, diagnosis, classification, and systems of supports. Am. J. Intellect. Dev. Disabil..

[2] Shree A., Shukla P.C. (2016). Intellectual Disability: Definition, classification, causes and characteristics. Learn. Comm. Int. J. Educ. Soc. Dev..

[3] Maulik P.K., Mascarenhas M.N., Mathers C.D., Dua T., Saxena S. (2011). Prevalence of intellectual disability: A meta-analysis of population-based studies. Res. Dev. Disabil..

[4] Olusanya B.O., Smythe T., Ogbo F.A., Nair M.K.C., Scher M., Davis A.C. (2023). Global prevalence of developmental disabilities in children and adolescents: A systematic umbrella review. Front. Public Health.

[5] Matson J.L., Matheis M., Estabillo J.A., Issarraras A., Peters W.J., Jiang X. (2019). Intellectual disability. Treatment of Disorders in Childhood and Adolescence.

[6] Perkins E.A., Small B.J. (2006). Aspects of cognitive functioning in adults with intellectual disabilities. J. Policy Pract. Intellect..

[7] Hwang A.W., Chao M.Y., Liu S.W. (2013). A randomized controlled trial of routines-based early intervention for children with or at risk for developmental delay. Res. Dev. Disabil..

[8] Guralnick M.J. (2017). Early intervention for children with intellectual disabilities: An update. J. Appl. Res. Intellect. Disabil..

[9] Patel D.R., Apple R., Kanungo S., Akkal A. (2018). Narrative review of intellectual disability: Definitions, evaluation and principles of treatment. Pediatr. Med..

[10] Querido J.S., Sheel A.W. (2007). Regulation of cerebral blood flow during exercise. Sports Med..

[11] Cefis M., Chaney R., Wirtz J., Méloux A., Quirié A., Leger C., Prigent-Tessier A., Garnier P. (2023). Molecular mechanisms underlying physical exercise-induced brain BDNF overproduction. Front. Mol. Neurosci..

[12] Carek P.J., Laibstain S.E., Carek S.M. (2011). Exercise for the treatment of depression and anxiety. Int. J. Psychiatry Med..

[13] Sriharisukesh N., Pailoor S., Sudharshanan S., Chathambally R. (2024). Effect of Yoga of Adaptive Yogasana Practice on the Flexibility and Psychomotor Variables in Intellectually Disabled Subjects. Indian. J. Community Med..

[14] Wang Y., Wu X., Chen H. (2023). Badminton Improves Executive Function in Adults Living with Mild Intellectual Disability. Int. J. Environ. Res. Public Health.

[15] Jacinto M., Antunes R., Monteiro D., Rodrigues F., Amaro N., Campos M.J., Ferreira J.P., Matos R. (2025). Examining the Effects of a 24-Week Exercise Program on Functional Capacity, Cognitive Capacity, and Quality of Life in Individuals With Intellectual and Developmental Disabilities. Adapt. Phys. Activ Q..

[16] Zhu G., Chen K., Ling C., Zhao P., Guo L. (2023). The Impact of physical activity on cognitive function in children and adolescents with intellectual disabilities: A meta-analysis. Int. J. Hum. Mov. Sports Sci..

[17] Page M.J., McKenzie J.E., Bossuyt P.M., Boutron I., Hoffmann T.C., Mulrow C.D., Shamseer L., Tetzlaff J.M., Akl E.A., Brennan S.E. (2021). The PRISMA 2020 statement: An updated guideline for reporting systematic reviews. BMJ.

[18] Chen Z., Zhou R., Liu X., Wang J., Wang L., Lv Y., Yu L. (2025). Effects of Aerobic Exercise on Blood Lipids in People with Overweight or Obesity: A Systematic Review and Meta-Analysis of Randomized Controlled Trials. Life.

[19] Viechtbauer W. (2010). Conducting meta-analyses in R with the metafor package. J. Stat. Softw..

[20] Assink M., Wibbelink C.J. (2016). Fitting three-level meta-analytic models in R: A step-by-step tutorial. Quant. Methods Psychol..

[21] Tyler C.J., Reeve T., Sieh N., Cheung S.S. (2024). Effects of heat adaptation on physiology, perception, and exercise performance in the heat: An updated meta-analysis. J. Sci. Sport. Exerc..

[22] Zhen K., Zhang S., Tao X., Li G., Lv Y., Yu L. (2022). A systematic review and meta-analysis on effects of aerobic exercise in people with Parkinson’s disease. NPJ Park. Dis..

[23] Higgins J.P., Thompson S.G., Deeks J.J., Altman D.G. (2003). Measuring inconsistency in meta-analyses. BMJ.

[24] Zhou Y., Ren H., Hou X., Dong X., Zhang S., Lv Y., Li C., Yu L. (2024). The effect of exercise on balance function in stroke patients: A systematic review and meta-analysis of randomized controlled trials. J. Neurol..

[25] Li G., You Q., Hou X., Zhang S., Du L., Lv Y., Yu L. (2023). The effect of exercise on cognitive function in people with multiple sclerosis: A systematic review and meta-analysis of randomized controlled trials. J. Neurol..

[26] Li G., Tao X., Lei B., Hou X., Yang X., Wang L., Zhang S., Lv Y., Wang T., Yu L. (2024). Effects of exercise on post-stroke cognitive function: A systematic review and meta-analysis of randomized controlled trials. Top. Stroke Rehabil..

[27] Atak E., Hajebrahimi F., Algun Z.C. (2023). The effect of Dual-Task balance exercises on cognitive functions among children with mild and borderline mental retardation: A randomized controlled trial. Eur. J. Physiother..

[28] Erkut A.O., Sunar C. (2025). Evaluation of the Effect of Trampoline and Movement Education Programs on the Development of Attention and Visual Perception in Preschool Children. J. Phys. Educ. Sport. Stud..

[29] Koper M., Lewandowska M., Rękosiewicz M. (2024). The effect of the Bilateral Integration exercise program on the cognitive functioning of pupils with moderate intellectual disabilities. Front. Psychiatry.

[30] Korkusuz S., Top E. (2023). Does the combination of physical activity and attention training affect the motor skills and cognitive activities of individuals with mild intellectual disability?. Int. J. Dev. Disabil..

[31] Lee I.H., Seo E.J., Lim I.S. (2014). Effects of aquatic exercise and CES treatment on the changes of cognitive function, BDNF, IGF-1, and VEGF of persons with intellectual disabilities. J. Exerc. Nutr. Biochem..

[32] Mero Piedra A.L., Pesthy O., Marton K. (2024). Effects of a physical education intervention on attention and inhibitory control in Ecuadorian children with intellectual disabilities. J. Intellect. Disabil..

[33] Nesrine B.M., Jarraya S., Caprioli L. (2025). The Effectiveness of a Mindfulness-Based Program in Improving Cognitive and Socio-Affective Skills Among Adolescents with Intellectual and Developmental Disabilities: A Randomized Controlled Study. Mindfulness.

[34] Song K.Y., An J.D. (2004). Premotor and motor reaction time of educable mentally retarded youths in a Taekwondo program. Percept. Mot. Ski..

[35] Tomporowski P.D., Ellis N.R. (1984). Effects of exercise on the physical fitness, intelligence, and adaptive behavior of institutionalized mentally retarded adults. Appl. Res. Ment. Retard..

[36] van Schijndel-Speet M., Evenhuis H.M., van Wijck R., van Montfort K.C., Echteld M.A. (2017). A structured physical activity and fitness programme for older adults with intellectual disabilities: Results of a cluster-randomised clinical trial. J. Intellect. Disabil. Res..

[37] Yildirim N.Ü., Erbahçeci F., Ergun N., Pitetti K.H., Beets M.W. (2010). The effect of physical fitness training on reaction time in youth with intellectual disabilities. Percept. Mot. Ski..

[38] Diz S., Gomes F., Santos S. (2021). Does physical activity improve adaptive behaviour, fitness, and quality of life of adults with intellectual disability?. Rev. Bras. De Cienc. Do Esporte.

[39] Hemayattalab R., Movahedi A. (2010). Effects of different variations of mental and physical practice on sport skill learning in adolescents with mental retardation. Res. Dev. Disabil..

[40] Firoozjah M.H., Firoozjah M.H., Sheikh M., Hemayattalab R., Shahrbanian S. (2019). The influence of environment potentiality (affordances) on motor development in 6–9 years old children with intellectual disability. Sport Sci. Health.

[41] Zhao J.G., Zeng X.T., Wang J., Liu L. (2017). Association between calcium or vitamin D supplementation and fracture incidence in community-dwelling older adults: A systematic review and meta-analysis. JAMA.

[42] Collins B.E., Hartmann T.E., Marino F.E., Skein M. (2024). The effect of a 12 week mixed-modality training intervention on the cardio-metabolic health of rotational shift workers. J. Sci. Sport. Exerc..

[43] Giustiniani A., Quartarone A. (2024). Defining the concept of reserve in the motor domain: A systematic review. Front. Neurosci..

[44] Knaepen K., Goekint M., Heyman E.M., Meeusen R. (2010). Neuroplasticity—Exercise-induced response of peripheral brain-derived neurotrophic factor: A systematic review of experimental studies in human subjects. Sports Med..

[45] Cunha C., Brambilla R., Thomas K.L. (2010). A simple role for BDNF in learning and memory?. Front. Mol. Neurosci..

[46] Hillman C.H., Logan N.E., Shigeta T.T. (2019). A review of acute physical activity effects on brain and cognition in children. Transl. J. Am. Coll. Sports Med..

[47] Filley C.M., Fields R.D. (2016). White matter and cognition: Making the connection. J. Neurophysiol..

[48] Tao J., Liu J., Chen X., Xia R., Li M., Huang M., Li S., Park J., Wilson G., Lang C. (2019). Mind-body exercise improves cognitive function and modulates the function and structure of the hippocampus and anterior cingulate cortex in patients with mild cognitive impairment. Neuroimage Clin..

[49] Srinivas N.S., Vimalan V., Padmanabhan P., Gulyás B. (2021). An overview on cognitive function enhancement through physical exercises. Brain Sci..

[50] Ma C.L., Ma X.T., Wang J.J., Liu H., Chen Y.F., Yang Y. (2017). Physical exercise induces hippocampal neurogenesis and prevents cognitive decline. Behav. Brain Res..

[51] Lafenetre P., Leske O., Wahle P., Heumann R. (2011). The beneficial effects of physical activity on impaired adult neurogenesis and cognitive performance. Front. Neurosci..

[52] Park J.W., Kwon Y.H., Lee M.Y., Bai D., Nam K.S., Cho Y.W., Lee C.H., Jang S.H. (2008). Brain activation pattern according to exercise complexity: A functional MRI study. NeuroRehabilitation.

[53] Douris P.C., Cottone J., Cruz P., Frosos N., Marino C., Singamenggala L., Shapiro J., Sousa A., Handrakis J.P., DiFrancisco-Donoghue J. (2023). The effects of externally paced exercise on executive function and stress in college-aged students. J. Sci. Sport Exerc..

[54] Deng J., Lei T., Du X. (2023). Effects of sensory integration training on balance function and executive function in children with autism spectrum disorder: Evidence from Footscan and fNIRS. Front. Psychol..

[55] Stroth S., Hille K., Spitzer M., Reinhardt R. (2009). Aerobic endurance exercise benefits memory and affect in young adults. Neuropsychol. Rehabil..

[56] Fox K.R. (1999). The influence of physical activity on mental well-being. Public Health Nutr..

[57] Ludyga S., Gerber M., Pühse U., Looser V.N., Kamijo K. (2020). Systematic review and meta-analysis investigating moderators of long-term effects of exercise on cognition in healthy individuals. Nat. Hum. Behav..

[58] Chen A.G., Zhu L.N., Yan J., Yin H.C. (2016). Neural basis of working memory enhancement after acute aerobic exercise: fMRI study of preadolescent children. Front. Psychol..

[59] Stern Y., MacKay-Brandt A., Lee S., McKinley P., McIntyre K., Razlighi Q., Agarunov E., Bartels M., Sloan R.P. (2019). Effect of aerobic exercise on cognition in younger adults: A randomized clinical trial. Neurology.

[60] Scholler V., Groslambert A., Grappe F., Grosprêtre S. (2023). General neural process in cycling exercise. J. Sci. Sport Exerc..

[61] Erostarbe-Pérez M., Reparaz-Abaitua C., Martínez-Pérez L., Magallón-Recalde S. (2022). Executive functions and their relationship with intellectual capacity and age in schoolchildren with intellectual disability. J. Intellect. Disabil. Res..

[62] Kim R., Lee T.L., Lee H., Ko D.K., Lee J.H., Shin H., Lim D., Jun J.S., Byun K., Park K. (2023). Effects of physical exercise interventions on cognitive function in Parkinson’s disease: An updated systematic review and meta-analysis of randomized controlled trials. Parkinsonism Relat. Disord..

[63] Lee M., Wee J., Dobbin N., Roman Q., Choong G. (2023). The Impact of Tournament Load on Neuromuscular Function, Perceived Wellness and Coach Ratings of Performance During Intensified Youth Netball Competition. J. Sci. Sport Exerc..

[64] Venegas-Sanabria L.C., Cavero-Redondo I., Martínez-Vizcaino V., Cano-Gutierrez C.A., Álvarez-Bueno C. (2022). Effect of multicomponent exercise in cognitive impairment: A systematic review and meta-analysis. BMC Geriatr..

[65] Jekauc D. (2015). Enjoyment during exercise mediates the effects of an intervention on exercise adherence. Psychology.

[66] Erickson K.I., Voss M.W., Prakash R.S., Basak C., Szabo A., Chaddock L., Kim J.S., Heo S., Alves H., White S.M. (2011). Exercise training increases size of hippocampus and improves memory. Proc. Natl. Acad. Sci. USA.

[67] Wagner G., Herbsleb M., de la Cruz F., Schumann A., Brünner F., Schachtzabel C., Gussew A., Puta C., Smesny S., Gabriel H.W. (2015). Hippocampal structure, metabolism, and inflammatory response after a 6-week intense aerobic exercise in healthy young adults: A controlled trial. J. Cereb. Blood Flow Metab..

[68] Piercy K.L., Troiano R.P., Ballard R.M., Carlson S.A., Fulton J.E., Galuska D.A., George S.M., Olson R.D. (2018). The Physical Activity Guidelines for Americans. JAMA.

[69] Shin I.S., Park E.Y. (2012). Meta-analysis of the effect of exercise programs for individuals with intellectual disabilities. Res. Dev. Disabil..

[70] Tsai C.L., Pan C.Y., Tseng Y.T., Chen F.C., Chang Y.C., Wang T.C. (2021). Acute effects of high-intensity interval training and moderate-intensity continuous exercise on BDNF and irisin levels and neurocognitive performance in late middle-aged and older adults. Behav. Brain Res..

[71] Schmolesky M.T., Webb D.L., Hansen R.A. (2013). The effects of aerobic exercise intensity and duration on levels of brain-derived neurotrophic factor in healthy men. J. Sports Sci. Med..

[72] Ellemberg D., St-Louis-Deschênes M. (2010). The effect of acute physical exercise on cognitive function during development. Psychol. Sport Exerc..

[73] Bull F.C., Al-Ansari S.S., Biddle S., Borodulin K., Buman M.P., Cardon G., Carty C., Chaput J.P., Chastin S., Chou R. (2020). World Health Organization 2020 guidelines on physical activity and sedentary behaviour. Br. J. Sports Med..

[74] Zhou R., Chen Z., Zhang S., Wang Y., Zhang C., Lv Y., Yu L. (2024). Effects of Exercise on Cancer-Related Fatigue in Breast Cancer Patients: A Systematic Review and Meta-Analysis of Randomized Controlled Trials. Life.

[75] Jahnsen R., Villien L., Stanghelle J.K., Holm I. (2003). Fatigue in adults with cerebral palsy in Norway compared with the general population. Dev. Med. Child. Neurol..

[76] Fuhrmann D., Knoll L.J., Blakemore S.J. (2015). Adolescence as a sensitive period of brain development. Trends Cogn. Sci..

[77] Zhang S., Gu B., Zhen K., Du L., Lv Y., Yu L. (2024). Effects of exercise on brain-derived neurotrophic factor in Alzheimer’s disease models: A systematic review and meta-analysis. Arch. Gerontol. Geriatr..

[78] Ma Y., Wang L., Li M., Wang T. (2020). Meta-analysis of the effects of exercise programs in improving the balance ability of children with intellectual disabilities. J. Intellect. Dev. Disabil..

