# Supplementary material for: Effects of Exercise on Cognitive Function in People with Intellectual Disabilities: A Systematic Review and Meta-Analysis of Randomized Controlled Trials
Source: Brain Sci. 2025 Nov 7;15(11):1203. doi: 10.3390/brainsci15111203 (PMC12651325; doi:10.3390/brainsci15111203)
Supplement: Supplementary file 1 [file brainsci-15-01203-s001.zip › brainsci-3925584-supplementary.pdf]

Supplemental material

Effects of exercise on cognitive function in people with intellectual disabilities: a systematic review and meta-analysis of randomized controlled trials

Figure S1. Results of Cochrane risk of bias tool.....................................2

Figure S2. Funnel plot...3

Figure S3. Sensitivity analysis results.. 4

Table S1. Search strategy.. 5

Table S2. Excluded studies list...6

Table S3. Characteristics of the studies included in this meta-analysis.... 9

Table S4. Results of meta-regression analysis...................................... 13

Table S5. GRADE summary of evidence.. 14

Table S6. Results of Egger's test... 15

Figure S1. Results of Cochrane risk of bias tool.

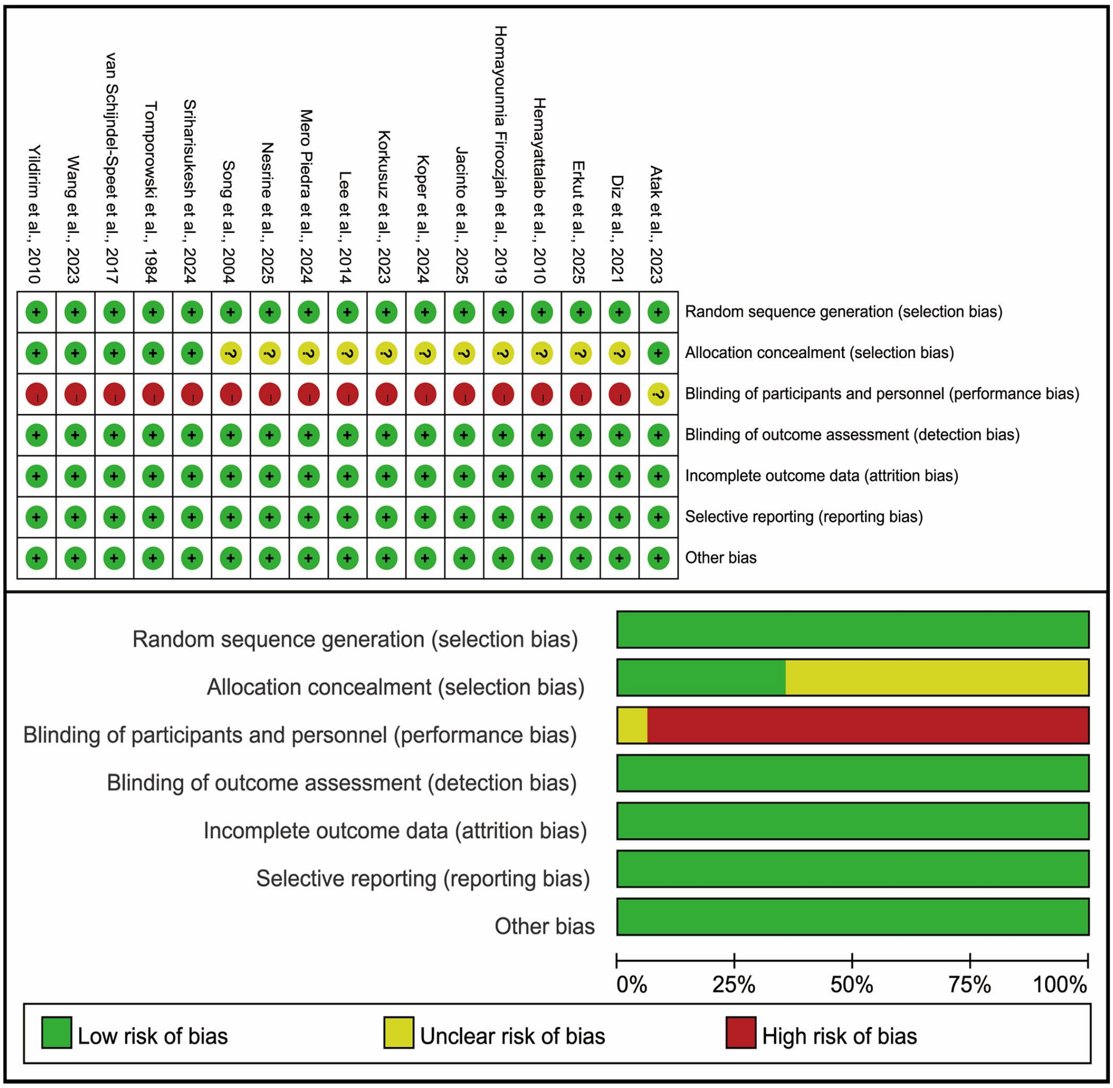

Figure S2. Funnel plot.

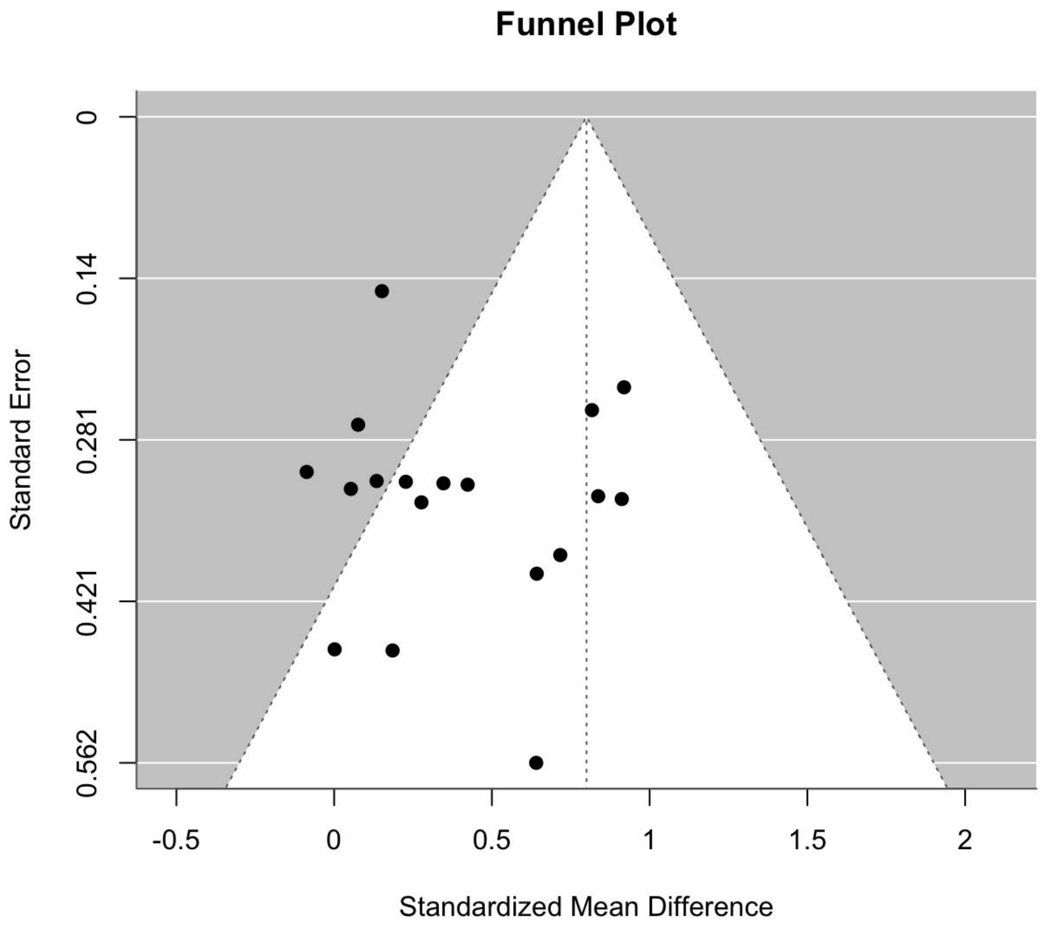

Figure S3. Sensitivity analysis results.

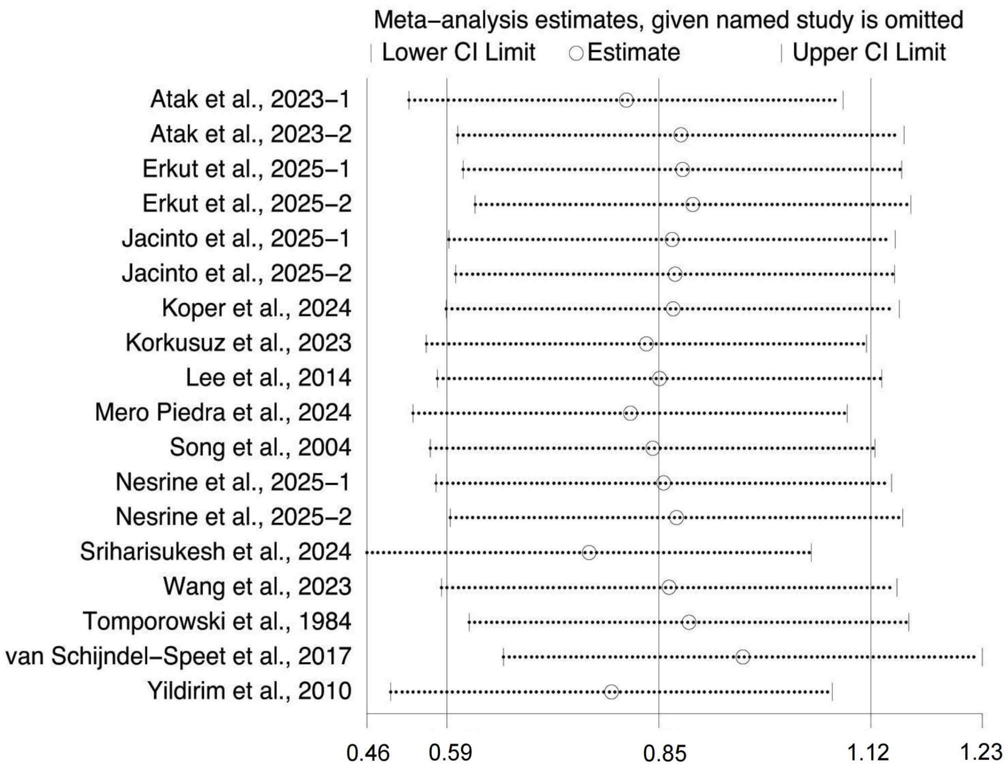

Table S1. Search strategy.

**Exercise search terms combined with 'OR'**
Physical exercise programs; Physical Therapy Modalities; Physical Therapy Modalities; Modalities, Physical Therapy; Modality, Physical Therapy; Physical Therapy Modality; Physiotherapy; Physical Therapy Techniques; Physical Therapy Technique; Techniques, Physical Therapy; Exercise Movement Techniques; Exercise Movement Techniques; Movement Techniques, Exercise; Exercise Therapy; Exercise Therapy; Therapy, Exercise; Exercise Therapies; Therapies, Exercise; Exercise, Physical; Exercises, Physical; Physical Exercise; Physical Exercises; Exercise, Isometric; Exercises, Isometric; Isometric Exercises; Isometric Exercise; Exercise, Aerobic; Aerobic Exercises; Aerobic Exercise; Resistance Training; Resistance Training; Training, Resistance; Strength Training; Training, Strength; Weight-Lifting Strengthening Program; Strengthening Program, Weight-Lifting; Strengthening Programs, Weight-Lifting; Weight Lifting Strengthening Program; Weight-Lifting Strengthening Programs; Weight-Lifting Exercise Program; Exercise Program, Weight-Lifting; Exercise Programs, Weight-Lifting; Weight Lifting Exercise Program; Weight-Lifting Exercise Programs; Weight-Bearing Strengthening Program; Strengthening Program, Weight-Bearing; Strengthening Programs, Weight-Bearing; Weight Bearing Strengthening Program; Weight-Bearing Strengthening Programs; Weight-Bearing Exercise Program; Exercise Program, Weight-Bearing; Exercise Programs, Weight-Bearing; Weight Bearing Exercise Program; Weight-Bearing Exercise Programs
**Intellectual disability search terms combined with 'OR'**
Disabilities, Intellectual; Intellectual Disabilities; Disability, Intellectual; Intellectual Development Disorder; Development Disorder, Intellectual; Development Disorders, Intellectual; Disorder, Intellectual Development; Disorders, Intellectual Development; Intellectual Development Disorders; Mental Retardation; Retardation, Mental; Idiocy; Mental Retardation, Psychosocial; Mental Retardations, Psychosocial; Psychosocial Mental Retardation; Psychosocial Mental Retardations; Retardation, Psychosocial Mental; Retardations, Psychosocial Mental; Deficiency, Mental; Deficiencies, Mental; Mental Deficiencies; Mental Deficiency
**Cognitive function search terms combined with 'OR'**
Cognitive function; Cognition; Cognitive Function; Cognitive Functions; Function, Cognitive; Functions, Cognitive; Executive Function; Executive Functions; Function, Executive; Functions, Executive; Executive Control; Executive Controls; Problem Solving; Perception; Perceptions; Memory; Attention; Attentions; Concentration; Concentrations; Learning

Table S2. Excluded studies list.

**Reasons**	**Title**
Absence of outcome indicators (*n* = 22)	1. Time Course Effects of Different Intensities of Running Exercise on Cognitive and Motor Performances in Individuals With Intellectual Disability<br>2. Psychological Benefits of Inclusive Soccer Program in Young Adults with and without Intellectual Disabilities<br>3. The effect of individual and paired Brailletonik exercises on balance and reaction time in children with intellectual disability<br>4. Effects of visual and auditory cognitive tasks on postural balance in adolescents with intellectual disability: A comparative analysis of trained versus non-trained individuals<br>5. Effects of exercise interventions on frailty in pre-maturely aging adults with intellectual disabilities- a preliminary study<br>6. Effects of Aquatic Training in Children with Autism Spectrum Disorder<br>7. Impact of a Service-Learning Program Using Soccer Training on the Emotional and Behavioral Problems of Children with Developmental Disabilities<br>8. Effects of a School-Based Physical Activity Intervention on Children with Intellectual Disability: a Cluster Randomised Trial<br>9. Effect of trampolines on physical activity in children with intellectual disabilities-concentration, steady state of brain, and pelvic tilt<br>10. Effect of a Cognitive Function and Social Skills-Based Digital Exercise Therapy Using IoT on Motor Coordination in Children with Intellectual and Developmental Disability<br>11. Going for a Walk: An Empirical Study of Route Learning Training and Its Effects on Mental and Physical Fitness in Patients with Korsakoff Syndrome<br>12. Impact of Acute Aerobic Exercise on Golf Putt Skill Learning in Adults With Intellectual Disabilities<br>13. Moderate cycling exercise enhances neurocognitive processing in adolescents with intellectual and developmental disabilities<br>14. Virtual reality as a leisure activity for young adults with physical and intellectual disabilities<br>15. Effects of Aquatic Exercise and Floor Curling on Balance Ability and Lower Limb Muscle Strength in Children with Intellectual Disabilities: a Pilot Study in China<br>16. The effectiveness of racket-sport intervention on visual perception and executive functions in children with mild intellectual disabilities and borderline intellectual functioning<br>17. Effects of moderate-intensity aerobic exercise combined with acupuncture on attention function of mentally-retarded adolescents: a randomised controlled trial

	18. Effects of alternated sport competition in perceived competence for adolescent males with mild to moderate mental retardation<br>19. The Effects of Virtual Zumba® on Functional Outcomes in Adults with Developmental Disabilities<br>20. Effects of integrated or segregated sport participation on the physical self for adolescents with intellectual disabilities<br>21. Effects of integrated sport participation on perceived competence for adolescents with mental retardation<br>22. Effectiveness of school-based physical activity programs in enhancing attention, academic performance, and social relationships among children with intellectual disabilities: evidence from Pakistani schools
Non-English articles (*n* = 17)	1. The Effects of Adapted Physical Activity Program on Perception of Spatial Relationship of Children with Intellectual and Developmental Disabilities<br>2. The Effect of Basketball Program on Anticipating Timing of Students with Mental Retardation<br>3. The Effect of Acute Moderate Exercise on Accuracy of Walking Performance Depending on Working Memory in individuals with Intellectual Disability<br>4. The Effect of Tai Chi Exercise on Attention and Brain activity in the Middle School Students with Intellectual Disability<br>5. The Effect of the After-school Rotating Exercise Program on Self-Determination and Sports Performance Abilities of Students with Mild Intellectual Disabilities<br>6. The Influence of Floorball Program on the physical fitness and attention concentration of students with Intellectual Disabilities<br>7. Developing and Applying a Community Dance Program to Improve Gross and Fine Motor Skills, Concentration, and Peer Play Interactions of Children with Intellectual Disabilities<br>8. Athletic competition: A means of improving the self-image of the mentally retarded adolescent?<br>9. Psychophysical effects of gymnastics for children with intellectual disabilities: physical and cognitive development<br>10. A study on the effect of trampoline movement applying psychological movement on attention focusing power and brain's stability of mentally handicapped children<br>11. Effects of Brain-Wave Training on Motor Competency in Individuals with Intellectual Disability<br>12. Impact of a multi-strategic group program on the level of daily life functioning of elderly persons with mild cognitive deficits living at home<br>13. The Effect of Participation in Integrated Physical Education Class on Brain Function Index of Children with Intellectual Disabilities<br>14. Comparison of Effectiveness of Motor-Working Memory Training and

	Perceptual-Motor Exercises on Digit Span and Letter – Number Sequencing in Educable Children with Intellectual Disabilities<br>15. How does the application of low impact out bound locomotor games affect the physical fitness of children with moderate disabilities?<br>16. Effect of Long-term Jump Rope Exercise on Attention and Self-determination in Intellectual Disabilities<br>17. Effects of 12-week Square-stepping Exercise on exercise perform capability and intellectual capability of children with developmental disability
Study protocol (*n* = 2)	1. Intellectual disability, exercise and aging: the IDEA study: study protocol for a randomized controlled trial<br>2. Effects of alternated basketball competition on perceived competence in adolescents with intellectual disabilities over a period of 13 months: A research note
Conference abstracts (*n* = 3)	1. The effects of physical exercise on reactive time and cognitive function in Tunisian mental deficient adolescents<br>2. Effect of trampolines on physical activity in children with intellectual disabilities-concentration, steady state of brain, and pelvic tilt<br>3. Multimodal interventions to delay dementia and disability in China (mind-China): baseline assessment
No control group (*n* = 3)	1. Impact of physical exercise on reactive time and cognitive function in mentally deficient adolescents<br>2. Acute effects of aerobic versus resistance exercises on cognitive function in individuals with intellectual disability<br>3. The Effect of a Video-Based Game Exercise Program on Motor Skills, Proprioception, and Cognitive Functions in Individuals With Intellectual Disabilities
Duplicate articles (*n* = 1)	1. Effects of running exercises on reaction time and working memory in individuals with intellectual disability

Table S3. Characteristics of the studies included in this meta-analysis.

Study	Sample size	Age(y)	Gender (male/female)	BMI	IQ	Intervention	Minutes per session	Frequency (times / week)	Duration (weeks)	Minutes per week	Cognitive domain	Cognitive task
Atak et al., 2023	Int1: 15<br>Int2: 15<br>Con: 15	Int1: 8.87 ± 1.45<br>Int2: 8.20 ± 1.52<br>Con: 8.93 ± 1.66	NR	Int1: 16.17 ± 1.85<br>Int2: 18.09 ± 3.32<br>Con: 17.73 ± 2.28	50-79	Int1: Cognitive training and DTBT<br>Int2: Standard balance and cognitive training	Int1: 40<br>Int2: 30	Int1: 2<br>Int2: 2	Int1: 12<br>Int2: 12	Int1: 80<br>Int2: 60	Memory<br>Speed<br>Executive	MOXO-Attention<br>MOXO-Timing<br>MOXO-Impulsivity<br>MOXO-Hyperactivity<br>WISC-R
Erkut et al., 2025	Int1: 16<br>Int2: 20<br>Con: 13	5-6	NR	NR	NR	Int1: Trampoline training program<br>Int2: Movement training program	Int1: 60<br>Int2: 60	Int1: 2<br>Int2: 2	Int1: 8<br>Int2: 8	Int1: 60<br>Int2: 60	Speed<br>Executive	FAT<br>FVPT
Jacinto et al., 2025	Int1: 7<br>Int2: 7<br>Con: 7	43.04 ± 11.18	11 / 10	NR	NR	Indoor training program	45	2	24	90	Memory<br>Speed<br>Executive	MMSE
Koper et al., 2024	Int: 13<br>Con: 14	Int: 13.23 ± 1.92<br>Con: 14.67 ± 2.08	Int: 8 / 5<br>Con: 8 / 6	NR	Int: 41.92 ± 5.54<br>Con: 40.07 ± 3.32	Bilateral integration school program	40	3	26	120	Memory<br>Executive	SB5-Fluid Reasoning<br>SB5-Knowledge<br>SB5-Quantitative Reasoning<br>SB5-Visual-Spatial Processing<br>SB5-Working Memory
Korkusuz et al., 2023	Int: 11<br>Con: 11	Int: 11.26 ± 2.03<br>Con: 11.33 ± 1.63	Int: 6 / 5<br>Con: 4 / 7	Int: 18.33 ± 4.43<br>Con: 17.73 ± 3.34	50-70	Cognitive movement, Running, Ball handling, Coordination training	100	2	14	200	Memory<br>Speed<br>Executive	d2-TN<br>d2-E1<br>d2-E2<br>d2-E

												d2-TN-E<br>d2-CP<br>d2-FR<br>BVRT
Lee et al., 2014	Int1: 5<br>Int2: 5<br>Con: 5	Int1: 15.60 ± 2.19<br>Int2: 15.80 ± 3.03<br>Con: 16.00 ± 1.87	NR	NR	NR	Int1: Aquatic training program<br>Int2: Aquatic training program, CES treatment	Int1: 50<br>Int2: 80	Int1: 3<br>Int2: 3	Int1: 12<br>Int2: 12	Int1: 150<br>Int2: 240	Memory<br>Speed<br>Executive	K-WAB
Mero Piedra et al., 2024	Int: 15<br>Con: 15	Int: 12.733 ± 1.438<br>Con: 12.6 ± 1.298	Int: 7 / 8<br>Con: 6 / 9	NR	NR	games	60	2	6	120	Speed<br>Executive	Vigilance Tasks<br>Distractor interference Tasks<br>Response inhibition Tasks
Song et al., 2004	Int: 10<br>Con: 10	Int: 18.8 ± 1.8<br>Con: 18.4 ±1.6	NR	NR	50-75	Taekwondo Program	45	3	28	135	Speed	Premotor RT<br>Motor RT
Nesrine et al., 2025	Int1: 15<br>Int2: 15<br>Con: 15	Int1: 15.67 ± 2.26<br>Int2: 16.40 ± 2.06<br>Con: 16 ± 2	Int1: 8 / 7<br>Int2: 8 / 7<br>Con: 8 / 7	NR	50-70	Int1: Mindfulness<br>Int2: PA program	Int1: 30<br>Int2: 45	Int1: 2<br>Int2: 2	Int1: 8<br>Int2: 8	Int1: 60<br>Int2: 90	Memory<br>Executive	The Digit Span Task<br>The Corsi Block - Tapping Test<br>The Zazzo Two - Sign Barrage Test
Sriharisukesh et al., 2024	Int: 20<br>Con: 20	Int: 20.4 ± 2.8<br>Con: 19.9 ± 3.76	Int: 19 / 11<br>Con: 17 / 13	Int: 15.81 ± 2.24<br>Con: 16.53 ± 2.21	NR	Yoga	45	6	5	270	Speed<br>Executive	WTT<br>FTT<br>SLCT
Wang et al., 2023	Int: 15<br>Con: 15	Int:36.00 ± 3.64<br>Con: 35.60 ± 4.32	Int: 10 / 5<br>Con: 10 / 5	Int: 24.87 ± 4.52<br>Con: 25.24 ± 3.84	50-69	Badminton	60	3	12	180	Memory<br>Speed<br>Executive	The Stroop test-inconsistent<br>The Stroop test-consistent<br>Back Test<br>Task Switching Test-no task<br>Task Switching Test-task

												Task Switching Test-flexibility ability
Tomporowski et al., 1984	Int: 20<br>Con: 22	Int: 17-39<br>Con: 15-35	NR	NR	Int: 26.77<br>Con: 27.00	Light calisthenics and flexibility exercises, Stationary bicycles, A rowing machine and weight-lifting equipment	180	5	28	900	Memory<br>Speed<br>Executive	Stanford-Binet (L-M)<br>Leiter International Performance Scale
van Schijndel-Speet et al., 2017	Int: 66<br>Con: 65	Int: 58.2<br>Con: 57.9	Int: 28 / 38<br>Con: 31 / 34	Int: 27.9<br>Con: 27.5	NR	Muscle strength, endurance, Balance and flexibility	45	3	32	135	Memory<br>Speed<br>Executive	DMR Cognitive subscale
Yildirim et al., 2010	Int: 25<br>Con: 25	Int: 14.52 ± 1.50<br>Con: 14.80 ± 1.29	Int: 19 / 6<br>Con: 20 / 5	NR	NR	Resistance training, Interval speed training	45	3	12	135	Speed	Newtest Reaction<br>Time Scale-Visual reaction<br>Newtest Reaction<br>Time Scale-Auditory reaction
Diz et al., 2021	Int: 8<br>Con: 8	Int: 40 ± 6.32<br>Con: 44.38 ± 6.66	Int: 3 / 5<br>Con: 4 / 4	NR	NR	Jumping in bows, Bypassing pins with a ball, Overcoming obstacles, Flexing	50	Biweekly	20	50 / Biweekly	Memory<br>Speed<br>Executive	PABS<br>TPMBO-2
Hemayattalab et al., 2010	Int1: 8<br>Int2: 8<br>Int3: 8<br>Con: 8	12-15	NR	NR	NR	Basketball free throw training; Mental practice	30	7	~ 4	210	Memory	NR

Homayounnia Firoozjah et al., 2019	Int: 25<br>Con: 25	Int: 7.4± 0.6<br>Con: 7.9 ± 0.8	NR	NR	53-73	Treadmill, Twist, Swing, Carousel, Slide, Bale pool	30-45	3	12	90-135	Memory<br>Speed<br>Executive	Stanford Binet<br>bot BOTMP

Abbreviation: Int, intervention groups; Con, control groups; NR, no record; BMI, body mass index; IQ, intelligence quotient; MOXO,MOXO attention test; FAT, Frankfurter Attention Test; FVPT, Frostig Visual Perception Test; MMSE, Mini-Mental State Examination; SB5, The Fifth Edition of Stanford–Binet Intelligence Subtests; d2, d2 test of attention; BVRT, Benton Visual Retention Test; K-WAB, Korean Western Aphasia Battery; WTT, Wall toss test; FTT, Finger tapping test; SLCT, Six letter cancellation task; PABS, Adaptive Behaviour Scale; TPMBO-2, The Bruininks-Oseretsky Test of Motor Proficiency, second edition; bot BOTMP, Bruininks–Oseretsky Test of Motor Proficiency;

Table S4. Results of meta-regression analysis.

_ES	Coef	Std. Err.	t	*p* > |t|	95% CI
**Cognitive domain**					
subgroup	0.1524989	0.2024611	0.75	0.453	-0.2502606, 0.5552584
_cons	0.3311808	0.4440289	0.75	0.458	-0.5521341, 1.214496
**Duration**					
subgroup	0.2289836	0.4211907	0.54	0.589	-0.6132393, 1.071207
_cons	0.3158253	0.7551198	0.42	0.677	-1.19413, 1.825781
**Frequency**					
subgroup	0.4229525	0.3731643	1.13	0.261	-0.3232357, 1.169141
_cons	1.0566209	0.6127322	0.09	0.927	-1.168613, 1.281855
**Session duration**					
subgroup	-0.8714439	0.3828438	-2.28	0.026	-1.636987, -0.1059004
_cons	2.179866	0.668196	3.26	0.002	0.8437259, 3.516007
**Weekly time**					
subgroup	1.696992	0.3829983	4.43	0.0001	0.9311393, 2.462844
_cons	-1.338768	0.4616059	-2.90	0.005	-2.261806, -0.4157295
**Age**					
subgroup	0.302698	0.4293073	0.71	0.483	-0.5557549, 1.161151
_cons	0.1885399	0.7737792	0.24	0.808	-1.358727, 1.735807

Abbreviation: Coef, coefficient; Std. Err, standard error; t, t-test statistic; *p*, probability; CI, confidence interval.

Table S5. GRADE summary of evidence.

Certainty assessment							No. of patients		Effect		Certainty	Importance
No. of studies	Study design	Risk of bias	Inconsistency	Indirectness	Imprecision	Other considerations	Exercise	Control	Relative (95% CI)	Absolute		
14	RCT	not serious	not serious	not serious	not serious	reporting bias	690	680	-	Hedges' *g* 1.01 higher (0.47 to 1.55 higher)	⊕⊕⊕⊕ high	-

Abbreviations: RCT, randomized controlled trial; CI, confidence interval; EF: executive function.

GRADE Working Group grades of evidence: ⊕⊕⊕⊕, High certainty: we are very confident that the true effect lies close to that of the estimate of the effect; ⊕⊕⊕○, Moderate certainty: we are moderately confident in the effect estimate: the true effect is likely to be close to the estimate of the effect, but there is a possibility that it is substantially different; ⊕⊕○○, Low certainty: our confidence in the effect estimate is limited: the true effect may be substantially different from the estimate of the effect; ⊕○○○, Very low certainty: we have very little confidence in the effect estimate: the true effect is likely to be substantially different from the estimate of effect.

Table S6. Results of Egger's test.

**Std_EFF**	**Coef.**	**Std. Err.**	**t**	***p* > |t|**	**95% CI**
Slope	-1.10836	0.2829247	-3.92	0.0001	-1.674103, -0.5426172
Bias	4.323023	0.7463617	5.79	0.0001	2.83058, 5.815465

Abbreviation: Coef, coefficient; Std. Err, standard error; t, *t*-test statistic; *p*, probability; CI, confidence interval.
